# Comparison of health‐promoting behaviours, eating behaviour patterns and perceived social support in normal‐weight and overweight pregnant women: An unmatched case–control study

**DOI:** 10.1002/nop2.447

**Published:** 2020-01-14

**Authors:** Sepideh Hajian, Azita Fathnezhad‐Kazemi

**Affiliations:** ^1^ Department of Midwifery & Reproductive Health Faculty of Nursing & Midwifery Shahid Beheshti University of Medical Sciences Tehran Iran; ^2^ Department of Midwifery, Tabriz Branch Islamic Azad University Tabriz Iran

**Keywords:** eating behaviour, health promotion, overweight, pregnancy, social support

## Abstract

**Aim:**

The interventions based on adopting a healthy lifestyle during pregnancy have conflicting results. This study aimed to compare health‐promoting, dietary patterns and social support in normal and overweight pregnant women.

**Design:**

An unmatched case–control design was used.

**Methods:**

A total of 360 pregnant women were selected using multistage cluster sampling and divided into two groups of normal and overweight cases. Data were collected using demographic and obstetrics characteristics, health‐promoting lifestyle, perceived social support and eating behaviour questionnaires.

**Results:**

The evaluation of the health‐promoting behaviours and dietary patterns demonstrated a significant difference between the mean of total scores and their subdomains including self‐actualization, nutrition, consumption of healthy and low‐fat foods, fast food and sweets, as well as emotional eating and accidental planning. There was no significant difference between the two groups about social support.

## 1 INTRODUCTION

Today, the health systems of countries encounter a major challenge known as obesity and overweight (Dodd et al., 2014) since the growing outbreak of obesity has led to an increase in the burden of chronic diseases and health‐related costs (Susan, Mallan, Callaway, Daniels, & Nicholson, 2017a). According to reports, one to two billion adults over the age of 20 years are obese and overweight (Bakhshi, Seifi, Biglarian, & Mohammad, 2012). Based on statistics, more than half of the women of reproductive age are overweight and start their pregnancy with a body mass index (BMI) above 25 (Dodd et al., 2014; O'Brien, Cramp, & Dodd, 2016).

On the other hand, pregnancy is a critical time for overweight and obesity in women (Thangaratinam et al., 2012; Tovar, Chasan‐Taber, Bermudez, Hyatt, & Must, 2010). Given the direct and indirect associations between overweight, obesity and high weight gain, in pregnant women and unfavourable pregnancy outcomes (e.g. increased mortality rate, diabetes, hypertension and birth complications), this issue is recognized as a major concern of healthcare providers (Gebler, Charuvastra, & Silver, 2015; Thangaratinam et al., 2012). Currently, due to the insufficiency of traditional approaches in the treatment of obesity and overweight, the main focus has been on the factors affecting the prevalence of obesity, such as the rapid increase in weight gain during childhood and pregnancy (Johnson, Gerstein, Evans, & Woodward‐Lopez, 2006).

Reports have indicated the effects of non‐genetic factors on the prevalence of obesity, and lifestyle is a considerable environmental factor in this regard (Ordovas, 2018). An unhealthy diet, immobility, socio‐economic factors and unfavourable social support are a part of an unhealthy lifestyle (Chen, Kuo, Chou, & Chen, 2007; Johnson & Schoeni, 2011; Shojaeezadeh, Estebsari, Azam, Batebi, & Mostafaee, 2008). Meanwhile, adopting health‐promoting behaviours and healthy lifestyle are considered as the determinants of individual and social health, factors for disease prevention and weight control. In this regard, social support can have a significant impact on the quality of life and adoption of health‐related behaviours due to a moderate effect on stressful events. In addition, it is a facilitating factor for continuing healthy behaviours (Kazemi & Hajian, 2018; Kazemi, Hajian, Ebrahimi‐Mameghani, & Khob, 2018; Susan, Mallan, Callaway, Daniels, & Nicholson, 2017b).

Despite the desire of pregnant women to show hygienic behaviours, the interventions based on adopting a healthy lifestyle, especially in terms of weight loss, have failed and yielded conflicting results (Susan et al., 2017a, 2017b). So that previous studies have demonstrated a significant difference in understanding healthy lifestyle of pregnant women with high and normal weight that exerted an impact on weight gain during pregnancy (Susan et al., 2017a, 2017b). Given the fact that pregnancy is an opportunity to affect the health of two generations, the necessary support must be provided through required services to improve the pregnant women's health and take appropriate measures for ideal weight gain during pregnancy (Johnson et al., 2006). Therefore, it is vital to realize the health‐related behaviours and needs of pregnant women, especially overweight and obese cases to enhance their health. We hypothesized that aspects of health‐promoting, nutritional behaviours and perceptions of social support could make a difference between the two groups. In this regard, the awareness about lifestyle would help design suitable interventions. With this background in mind, this study aimed to compare the health‐promoting behaviours, nutritional–behavioural patterns and perceived social support among the two groups of overweight and normal‐weight women.

## 2 METHODS

### 2.1 Study design and participants

This unmatched case–control study was conducted during the first 6 months of 2017 in Tabriz, Iran. The inclusion criteria included the Iranian nationality, Tabriz residency, singleton pregnancy, ability to read and write in Persian, age range of 18–40 years, BMI registered within the range of 18.5–24.9 as a control and 25–29.9 as a case groups before pregnancy based on medical records, no experience of severe psychological crises over the past 6 months (declared by the participants), no known medical disorders or problems and obstetric risk factors during and before pregnancy based on medical records approved by the physician or midwife at the centre. The exclusion criteria were lack of willingness to participate in the study and incomplete questionnaires.

### 2.2 Sample size and sampling

The power analysis method was used to calculate the sample size. Since the largest sample size was obtained by considering health‐promoting behaviours, this result was applied to estimate the sample size. In this regard, considering the results of a study carried out by Al‐Kandari, Vidal, and Thomas in Kuwait (2008) the mean scores of health‐promoting behaviours in normal‐weight and overweight cases were 2.8 (0.53) and 2.6 (0.49), respectively. The effect size was calculated as 0.38 according to the equation. However, the sample size was calculated as 120 for each group considering 80% test power and 0.05 Type I error. In the light of the effect size of 1.5, the final sample size was estimated as 180 for each group. It should be noted that the G*Power software was exploited to calculate the sample size.

After the approvals were obtained from the authorities of the healthcare centres, a number of centres by multistage cluster sampling were randomly selected from 11 branches existing in the city, including 20 healthcare complexes and 87 healthcare centres using the Randomizer software. In total, 36 healthcare centres were selected. Afterwards, the suitable sample size was calculated and determined for each centre or according to the main sample size (*N* = 360) using quota sampling method and based on demographic characteristics of the centres.

Then, the list of all qualified pregnant women covered by each unit was extracted. Moreover, the names of the people were put in columns with numbers and randomly selected using computer and the Randomizer software. The lack of meeting the criteria for entering the study led to the replacement of the participant with a person randomly chosen from the list. The sampling continued until reaching the estimated sample size for both groups. It is noteworthy that the selection of the cases introduced as the main individuals on the list was prioritized based on the quota of the centre.

### 2.3 Instruments

#### 2.3.1 Socio‐demographic and obstetrics characteristics

It consists of the demographic variables of pregnant women containing age, educational level, occupational status of pregnant women and their spouses, self‐assessment of household economic status, as well as obstetrics characteristics, including the first day of the last menstruation, probable due date, gestational age based on first trimester ultrasound, number of pregnancies and childbirths, as well as height and weight before pregnancy.

#### 2.3.2 Health‐promoting Lifestyle‐II Questionnaire

It contains 52 items assessing six dimensions of nutrition (nine items), exercise (eight items), accountability regarding health (nine items), stress management (eight items), interpersonal support (nine items) and self‐actualization (nine items). All the items are scored based on a four‐point Likert scale (1 = never, 2 = sometimes, 3 = often, 4 = always). The total score for these behaviours is within the range of 52–208 (Walker, Sechrist, & Pender, 1987). The Persian version of this tool, on the population as a whole (i.e. men and women), has been evaluated in previous studies in terms of validity and reliability, and the Cronbach's alpha coefficients for the total tool and its dimensions were obtained as 0.82 within the range of 0.64–0.91, respectively. In addition, the questionnaire had sufficient stability (0.89) (Khazaeian, Kariman, Ebadi, & Nasiri, 2018; Zeidi, Hajiagha, & Zeidi, 2012).

#### 2.3.3 The multidimensional scale of perceived social support

This is a social support questionnaire designed by Zimet et al. that encompasses 12 items scored based on a Likert scale. The questionnaire evaluates three domains of perceived support from the family (four items), perceived support from family members and acquaintances (four items), and perceived support from friends (four items). The items are scored based on a seven‐point Likert scale from “completely disagree” (score: 1) to “completely agree” (degree: 7) where the minimum and maximum scores are 12 and 84, respectively (Zimet, Dahlem, Zimet, & Farley, 1988). Its validity and reliability were confirmed in Iran; its validity was confirmed through content analysis and reliability in various studies was estabilished using Cronbache's Alpha cofficient (α=0.86‐0.9 for the subscales and 0.86 for the whole instrument)(Bagherian‐Sararoudi, Hajian, Ehsan, Sarafraz, & Zimet, 2013; Sharifi et al., 2017).

#### 2.3.4 Eating behaviour pattern questionnaire

It contains six dimensions of low‐fat eating (11 items), convenience snack foods (fast food) and sweets (10 items), emotional eating (eight items), accidental planning (six items), meal skipping (seven items) and cultural/lifestyle behaviours (nine items). All the items were scored based on a five‐point Likert scale from completely agree to completely disagree (Schlundt, Hargreaves, & Buchowski, 2003). According to a study, the Persian version of the tool in the women has an appropriate validity and reliability (Dehghan, Asghari‐Jafarabadi, & Salekzamani, 2015).

### 2.4 Data collection

The subjects were chosen after the referral to healthcare centres and investigation of the pregnant women's medical files. The cases with BMI 18.5–24.9 before pregnancy and a group of women with BMI within the range of 25–29.9 during the same period were contacted through phone calls or in‐person consultation. First, the researcher explained the objectives of the study and requested the women to determine a time and date for referring to the healthcare centre to complete the questionnaire in case of willingness to participate in the project. On referral to the centres, in addition to the primary evaluations by the researcher, a written informed consent was obtained from the subjects. Following that, the study questionnaires were completed by each participant in one of the empty rooms of the centre.

### 2.5 Analysis

Data analysis was performed in SPSS software (version 21) using descriptive statistics to adjust the frequency tables and determine the central indexes, as well as the distribution of study variables to describe the features of the research units, health‐promoting behaviours, social support and nutritional behaviours. Furthermore, the data were analysed using analytical statistics, including chi‐square and independent *t* test (to compare the quantitative variables) and logistic regression analysis. The normality of quantitative data was measured based on kurtosis and skewness, all of which were normal. All the statistical tests were two‐sided, using a significance level of *p* < .05. It should be noted that the Strengthening the Reporting of Observational Studies in Epidemiology (STROBE) standard was used to report this article.

### 2.6 Ethics approval and consent to participate

The University Research Ethics Committee (ID.1395.498. as part of a PhD dissertation) approved this study. After the researchers had explained the purpose and content of the study, written informed consent was obtained from all participants.

## 3 RESULTS

In the present study, 17 women had no desire to participate in the study and 25 questionnaires were incomplete all of which were excluded from the study and the sampling continued until reaching 180 participants.

### 3.1 Characteristics of participants

The obtained results of the present study indicated that the mean age of participants was 27.56 (*SD* 5.09) years and most participants (76.1%) had diploma or lower educational level and 90% were housewives. According to the number of pregnancies, most pregnant women were nulliparous 172 (48.6%) and 126 (35%) second pregnancy. Data analysis demonstrated that there was no significant difference in the demographic characteristics of the two groups, with the exception of maternal age, number of pregnancies and educational level of the spouse (Table 1).

**Table 1:** Socio‐demographic and obstetrics characteristics of pregnant women

Variable	Normal‐weight group	Overweight group	*p*‐value
	(*N* = 180)	(*N* = 180)	
	Mean (*SD*)	Mean (*SD*)	
Maternal age	26.91 (4.8)	28.07 (4.9)	.025
Number of pregnancies	1.54 (0.8)	1.91 (0.9)	<.001
Gestational week	23.38 (8.2)	24.63 (9.1)	.175
BMI	22.49 (1.5)	28.05 (1.4)	

Variable	Normal‐weight group	Overweight group	*p*‐value
	*N* (%)	*N* (%)	
Maternal educational level			
Diploma or below diploma degrees	129 (71.1)	134 (73.4)	0.672
Academic degrees	51 (28.3)	46 (26.6)	
Maternal occupational status			
Housewife	162 (90.0)	164 (91.1)	09.19
Employed	18 (10.0)	16 (9.9)	
Educational level of spouse			
Diploma or below diploma degrees	125 (69.9)	148 (83.6)	.003
Academic degrees	55 (30.1)	29 (16.4)	
Occupational status of spouse			
Unemployed	4	5 (2.8)	.117
Employed	40 (22.3)	25 (13.9)	
Self‐employed	136 (75.6)	150 (83.3)	
Income level			
Less than sufficient	57 (31.7)	48 (26.7)	.378
Sufficient	122 (67.8)	131 (72.7)	
More than sufficient (ability to save money)	1 (0.6)	1 (0.6)	

### 3.2 Comparison of Health‐promoting Behaviours in Case and Control Groups

According to the results, the mean of total score of health‐promoting lifestyle in women with normal BMI and overweight women was 371.51 and 132.89, respectively. The highest mean score was related to the flourishment and nutrition field, and the lowest mean score was associated with stress management and exercise that was true in both groups. In sum, the comparison of scores of different dimensions indicated a significant difference between the two groups in terms of self‐actualization and nutrition dimensions, in a way that the mean scores of these domains were lower in the group with overweight BMI, compared to that of the control group (*p* < .05) (Table 2).

**Table 2:** Comparison of mean score of dimensions of health‐promoting behaviours in pregnant women

Dimensions of health‐promoting behaviours	Normal‐weight group	Overweight group	*p*‐value^a^
	Mean (*SD*)	Mean (*SD*)	
Interpersonal support	23.52 (3.72)	22.88 (4.26)	.135
Accountability	22.90 (4.45)	22.08 (4.63)	.087
Exercise	17.08 (4.11)	16.34 (4.15)	.087
Self‐actualization	27.35 (4.84)	26.32 (4.89)	.046
Nutrition	26.60 (4.09)	25.73 (4.30)	.051
Stress management	20.03 (3.56)	19.58 (3.98)	.258
Total score	137.51 (19.33)	132.89 (20.50)	.029

a Independent *t* test

One‐variable regression analysis showed that one‐point increase in the mean score of self‐actualization and nutrition led to the 5% reduction of overweight chance. In total, one‐point score increase in all aspects of health promotion resulted in a 2% decrease in weight gain chance (Table 3).

**Table 3:** Results of logistic regression analysis for evaluation of effect of health‐promoting behaviours on body mass index

Dimensions of health‐promoting behaviours	Odd ratio (95% confidence interval)	*p*‐value
Interpersonal support	0.961 (0.912–1.012)	.135
Accountability	0.961 (0.918–1.006)	.087
Exercise	0.957 (0.910–1.007)	.087
Self‐actualization	0.957 (0.917–0.999)	.046
Nutrition	0.952 (0.906–1.000)	.051
Stress management	0.969 (0.917–1.024)	.258
Total score	0.988 (0.978–0.999)	.029

### 3.3 Comparison of nutritional–behavioural patterns between case and control groups

In addition, the results of data analysis indicated that the total score of nutritional–behavioural patterns between the two normal‐weight and overweight groups was 156 and 160, respectively. While the highest score in the two groups was related to the consumption of low‐fat and healthy foods, and cultural and lifestyle behaviours, the lowest score was related to accidental planning and skipping the meal. Regarding nutritional–behavioural patterns, the results were indicative of a significant difference between the groups considering the total score and score of dimensions of consuming low‐fat and healthy foods, fast food and sweets, as well as emotional eating and accidental planning (*p* < .001) (Table 4).

**Table 4:** Comparison of mean score of nutritional–behavioural patterns in pregnant women

Dimensions of nutritional–behavioural patterns	Normal‐weight group	Overweight group	*p*‐value^a^
	Mean (*SD*)	Mean (*SD*)	
Consumption of low‐fat and healthy foods	41.04 (4.99(	39.12 (5.03)	<.001
Fast food and sweets	23.84 (5.17)	26.63 (5.25)	<.001
Emotional eating	23.11 (4.17)	24.92 (3.92)	<.001
Accidental planning	18.52 (3.05)	19.43 (2.93)	.004
Skipping a meal	22.40 (3.39)	22.38 (3.50)	.976
Cultural behaviours and lifestyle	27.55 (3.42)	27.91 (4.46)	.395
Total score	156.50 (15.22)	160.54 (15.85)	.015

a Independent *t* test.

One‐variable regression analysis demonstrated that the increase of one score in domain of fast food, sweets and emotional eating was associated with a probability of weight gain with the 1:11 ratio. In addition, the increase of one score in domain of accidental planning increased the chance of weight gain by 1.1 times (Table 5).

**Table 5:** Results of logistic regression analysis for evaluation of effect of nutritional–behavioural patterns on body mass index

Nutritional–behavioural patterns	Odd ratio (95% confidence interval)	*p*‐value
Consumption of low‐fat and healthy foods	0.926 (0.887–0.967)	<.001
Fast food and sweets	1.110 (1.063–1.159)	<.001
Emotional eating	1.118 (1.059–1.180)	<.001
Accidental planning	1.108 (1.031–1.190)	.005
Skipping a meal	0.999 (0.941–1.061)	.976
Cultural behaviours and lifestyle	1.023 (0.971–1.078)	.394
Total score	1.017 (1.003–1.031)	.016

### 3.4 Comparison of perceived social support in case and control groups

The evaluation of the overall score of perceived social support and the relevant domains in the two normal‐weight and overweight groups showed no significant difference. In addition, the highest mean was related to the social protection of the family and special individuals, while the lowest mean score was related to the social support of friends (Table 6).

**Table 6:** Comparison of mean score and level of social support of pregnant women

Social support	Normal‐weight group	Overweight group	*p*‐value^a^
	Mean (*SD*)	Mean (*SD*)	
Total score of social support	6.097 (14.10)	59.65 (14.85)	.389
Social support for special individuals	21.99 (5.96)	21.30 (5.83)	.269
Social support of friends	17.05 (6.19)	16.37 (6.20)	.300
Social support of family	21.93 (5.67)	21.92 (5.97)	.985

Levels of social support	Normal‐weight group	Overweight group	*p*‐value^a^
	*N* (%)	*N* (%)	
Low	32 (51.6)	30 (48.4)	.220
Moderate	85 (45.9)	100 (54.1)	
High	63 (56.2)	49 (43.8)	

a Independent *t* test.

## 4 DISCUSSION

In the present study, the health‐promoting behaviours, nutritional patterns and perceived social support were compared between the two groups of normal‐weight and overweight pregnant women. According to the obtained results, there was a significant difference between the subjects in terms of adopting a healthy lifestyle and performing health‐promoting behaviours, including nutritional pattern. Nonetheless, no significant difference was observed between the two groups regarding perceived social support.

The evaluation of the mean scores of total health‐promoting behaviours in both overweight (132.89) and normal‐weight (137.51) groups showed that both groups had a moderate level in terms of adopting such behaviours, based on the results of other studies (Baheiraei et al., 2011; Gokyildiz, Alan, Elmas, Bostanci, & Kucuk, 2014; Malakouti, Sehhati, Mirghafourvand, & Nahangi, 2015). In this regard, the findings of this study are comparable to the results of similar studies carried out in Iran and other countries, with the exception of two studies conducted by Taopia in Thailand and Onat in Turkey that reported a better overall score, compared to those of other studies (Onat & Aba, 2014; Thaewpia, Howland, Clark, & James, 2013). This lack of consistency between the aforementioned studies and the present study might be attributed to the impact of the factors, such as cultural differences and pregnancy age, on the studied subjects. Generally, the assessed cases in the mentioned studies, all of the women were in their second trimesters of pregnancy but in our study only 182 (52.5%) were in their second trimesters. It seems that during this period, women had a more stable condition, compared to other pregnancy trimesters.

In the present study, it was concluded that overweight pregnant women obtained a lower overall score in adopting health‐promoting behaviours, compared to the subjects in the control group. In addition, this difference was statistically considered significant. In this respect, the results of a study carried out by Cho et al. are in line with the findings of this study. In the aforementioned study, the status of health‐promoting behaviours was evaluated in overweight and obese women within the age range of 18–65 years and it was reported that increased level of BMI was associated with the decreased total score of health‐promoting behaviours (Cho, Jae, Choo, & Choo, 2014). Moreover, the evaluation of status of health‐promoting behaviours in other demographic groups, such as nursing students, suggested that fewer overweight people participated in such activities, compared to the individuals with normal weight that is in line with the findings of the present study (Al‐Kandari et al., 2008; Chen et al., 2007). In this regard, according to a report by Susan et al. (2017a), understanding the lifestyle behaviours in different weight groups is significantly different and individual factors (e.g. perceived control of their behaviour) are a determining factor to show health behaviours.

In addition, the obtained results demonstrated that while women in both groups received high scores regarding self‐actualization and nutrition, they obtained a low mean score in exercise and stress management dimensions. Nonetheless, overweight women received lower scores in terms of self‐actualization and proper nutrition, compared to the subjects in the control group. The results of studies conducted in different parts of Iran indicated that pregnant women obtained the highest scores regarding spiritual growth (self‐actualization) and nutritional status, while they obtained the lowest score in the domains of stress management and physical activity (Basharpoor, Heydarirad, Atadokht, Daryadel, & Nasiri‐Razi, 2015; Mahmoodi et al., 2015; Malakouti et al., 2015). The aforementioned results are consistent with the findings of the present study.

Furthermore, the review of studies conducted in other countries (e.g. Jordan and Turkey) suggested that pregnant women received high scores in self‐actualization, social support and accountability fields while they obtained moderate and low scores in the domains of nutrition, as well as stress management and physical activity, respectively (Gharaibeh, Al‐Ma'aitah, & Al Jada, 2005; Gokyildiz et al., 2014; Kavlak et al., 2013). This inconsistency between the results might be due to the environmental and cultural differences. Moreover, the results of a study carried out by Nies, Buffington, Cowan, and Hepworth (1998) in the United States are not consistent with the findings of the present study. In the aforementioned study, overweight non‐pregnant women achieved lower scores in all aspects of health‐promoting behaviours, compared to normal‐weight individuals.

In addition, the assessment of the status of health‐promoting behaviours in Taiwanese adolescents showed a low score in terms of social support, accountability and physical activity (Chen et al., 2007). This inconsistency in the results might be related to the diversity of the subjects regarding the age, gender and lack of pregnancy since the specific pregnancy conditions of women in the present study can affect the adoption of behaviours. In general, pregnant women tend to change their behaviours to achieve the desired outcomes and they are more likely to display hygienic behaviours. Regarding the results, one of the important problems in overweight women was the low scores in the dimensions of spirituality and self‐actualization. According to the findings of other studies, increasing the conception of spirituality and self‐actualization is associated with the reduction of high‐risk behaviours in pregnant women and addressing spirituality by decreasing stress improves the health condition during pregnancy. Proper interventions in this domain can be helpful. Another important issue was obtaining a low score in the physical activity dimension in both groups. However, the factors, such as constraints for women in society and spent time for family‐related tasks, can be effective; in this regard, pregnancy and belief in more rest during this period can be an important factor in getting the lowest score, compared to other dimensions of health‐promoting behaviours. Given the fact that physical activity during pregnancy is associated with the improvement of maternal and neonatal outcomes and control of weight gain, planning is required for proper interventions in this respect.

The comparison of the nutritional–behavioural patterns indicated a significant difference between the two groups in this regard. Furthermore, this comparison showed a correlation between eating pattern and BMI, in a way that overweight women had improper nutritional patterns, such as the consumption of fast food and sweets, and emotional eating with no planning and consumed less healthy and low‐fat foods, compared to the subjects in the control group. These findings are in line with the results of the present study (Cardon et al., 2016; Chitsaz, Javadi, Lin, & Pakpour, 2017). Other studies have also reported the association between the overall score of nutritional behaviours and BMI (Bashirian, Jalily, & Barati, 2016). A review of the related literature revealed that overweight people usually have an improper diet and deal with more nutritional deficiencies, compared to normal‐weight individuals. In addition, the unbalanced reception of macronutrients and micronutrients is higher in these subjects (Groth & Morrison‐Beedy, 2013; Hui et al., 2012).

As it was reported by Kolko, Emery, Marcus, and Levine (2017), a significant percentage of overweight pregnant women lose their eating control in pregnancy, compared to pre‐pregnancy period and emotional eating increased in these cases. Shloim, Rudolf, Feltbower, Blundell‐Birtill, and Hetherington (2018) marked a significant relationship between BMI and emotional eating and increased fast food consumption. On the other hand, in the aforementioned study, no association was observed between dietary restrictions and BMI during pregnancy. Similarly, no significant relationship was noticed between BMI and skipping a meal in the present study. According to the literature, it was suggested that although pregnant women restrict the consumption of certain substances, such as alcohol, tea and caffeine during pregnancy and try to change their diet, their consumption of healthy and vital foods (e.g. fruits, vegetables and the foods containing protein and low fat) was not optimal that was more noticeable in overweight subjects, compared to that of normal‐weight cases (Crozier et al., 2009). As it was observed in the present study, despite the fact that pregnant women obtained high scores regarding health‐promoting behaviours in nutrition domain, overweight cases did not follow a suitable diet, which can be due to a lack of awareness about food choice. Based on the evidence, it was shown that various factors, such as internal (self‐related) and external factors, including control perception, demographic characteristics, external support and health policies, can be effective in adopting healthy behaviours, such as nutritional behaviours.

According to the obtained results of the present study, however, the overall score and various dimensions of perceived social support were lower in overweight individuals, compared to those of normal‐weight subjects, and this difference was not statistically significant. In addition, low scores were obtained in both groups in terms of understanding friends’ support. These findings are comparable to the results of other studies. Based on the results of a study carried out by Susan et al. (2017a) in Australia, no difference was observed between the two groups of overweight and normal‐weight pregnant women in terms of perceived social support. Similarly, Johnson et al. (2006) noticed no association between perceived social support and the BMI of African American women with different weights. However, the results of other studies are inconsistent with the findings of the present study (Harrison, Teede, Kozica, Zoungas, & Lombard, 2017; Linder, Sacheck, Noubary, Nelson, & Freeman, 2017). In a study conducted by Harrison et al. (2017), a reverse relationship was observed between perceived social support, and BMI and weight gain in women, which can be attributed to the fact that over 65% of the studied cases were overweight. In addition, Linder et al. (2017) marked a lower score of perceived social support in children with different classes of BMI, especially family support. The observed difference in the research community is in terms of age. It can also be stated that pregnant women are usually supported by their relatives due to their particular conditions. The important issue is that while no significant difference was observed between the two groups in the social support dimension of health‐promoting behaviours, as previously mentioned, the results of studies performed in the country have shown moderate scores in social support. Meanwhile, the findings of studies from other parts of the world have demonstrated high scores obtained by pregnant women in this domain. Since a review of the literature suggests the facilitating effect of social support perception on adopting a healthy lifestyle (Stark & Brinkley, 2007; Sui, Turnbull, & Dodd, 2013; Walker, Cooney, & Riggs, 1999), it seems that further studies are required to take measures in this regard.

## 5 LIMITATIONS OF THE STUDY

There are some limitations despite the fact that the present study is the first one to examine the differences in health‐promoting lifestyle in overweight and normal‐weight pregnant women. Self‐report was one of the drawbacks of this study. In addition, the lack of reliable and valid questionnaires for the pregnant women was another study limitation. Therefore, the obtained results of the present study can only be generalized to overweight and normal‐weight cases and not to obese and low‐weight individuals. Although the sampling did not match, we used random sampling. Furthermore, since different changes occur in various pregnancy trimesters, it is suggested to perform and compare reviews every 3 months between matched groups.

## 6 CONCLUSION

The identification and better perception of the status of health‐related behaviours, such as how to adopt health behaviours, especially nutrition and social support perception, as a moderator of behaviours for positive support and health improvement are significantly crucial. The obtained results of the present study showed that while overweight pregnant women achieved lower scores in adopting health‐promoting behaviours, both groups obtained moderate scores. They especially gained lower scores in the dimensions of stress management and physical activity. In addition, overweight women had unfavourable conditions in most domains, and despite being pregnant, they had an inappropriate food pattern. Both groups had a moderate condition in terms of social support, and the support from friends was reported at the lowest level. Therefore, according to the obtained results of this study, it is recommended to integrate healthy plans in future health‐promoting interventions to achieve optimal results.

## CONFLICT OF INTEREST

The authors declare that they have no competing interests.

## AUTHOR CONTRIBUTIONS

AF‐K contributed to development of the concept, collected data, analysed data and wrote the draft and final article. SH contributed to development of concept and reviewed the draft and final article. All authors read and approved the final manuscript.
